# The Effector Domain of the Influenza A Virus Nonstructural Protein NS1 Triggers Host Shutoff by Mediating Inhibition and Global Deregulation of Host Transcription When Associated with Specific Structures in the Nucleus

**DOI:** 10.1128/mBio.02196-21

**Published:** 2021-09-07

**Authors:** Wolfgang Nacken, André Schreiber, Dörthe Masemann, Stephan Ludwig

**Affiliations:** a Institute of Virology (IVM), University of Muenster, Muenster, Germany; b University Hospital Muenster, Muenster, Germany; c Interdisciplinary Center of Clinical Research (IZKF), Medical Faculty of Westfaelische Wilhelms University of Muenster, Muenster, Germany; The Peter Doherty Institute for Infection and Immunity

**Keywords:** influenza virus, NS1, host shutoff, transcription, transcriptional repression

## Abstract

Host shutoff in influenza A virus (IAV) infection is a key process contributing to viral takeover of the cellular machinery and resulting in the downregulation of host gene expression. Analysis of nascently transcribed RNA in a cellular model that allows the functional induction of NS1 demonstrates that NS1 suppresses host transcription. NS1 inhibits the expression of genes driven by RNA polymerase II as well as RNA polymerase I-driven promoters, but not by the noneukaryotic T7 polymerase. Additionally, transcriptional termination is deregulated in cells infected with wild-type IAV. The NS1 effector domain alone is able to mediate both effects, whereas NS1 mutant GLEWN184-188RFKRY (184-188) is not. Overexpression of CPSF30 counteracts NS1-mediated inhibition of RNA polymerase II-driven reporter gene expression, but knockdown of CPSF30 expression does not attenuate gene expression. Although NS1 is associated with nuclear chromatin, superresolution microscopy demonstrates that NS1 does not colocalize with genomic DNA. Moreover, NS1 mutants and NS1 fusion proteins, unable to associate with nuclear chromatin and displaying an altered subcellular distribution are still able to attenuate reporter gene expression. However, tethering NS1 artificially to the cytoskeleton results in the loss of reporter gene inhibition. A NS1 deficient in both native nuclear localization signals (NLS) is able to inhibit gene expression as effective as wild-type NS1 when a synthetic NLS relocates it to specific structures of the nucleus. Colocalization experiments and reporter gene cotransfection experiments with a NS1 fusion guiding it to nuclear speckles suggest that the presence of NS1 in nuclear speckles seems to be essential for host shutoff.

## INTRODUCTION

Influenza A viruses (IAVs), unlike most other RNA viruses, replicate in the nucleus, a feature that places evolutionary pressure on a number of viral proteins to cooperate or interfere with host chromatin-based regulatory processes in infected cells (1, 2). IAV encodes the nonstructural protein NS1, which antagonizes host antiviral responses (3). This occurs through multiple mechanisms, including inhibition of virus sensing (4) and suppression of host functions that are detrimental to the virus, such as host translation (5) and anti-inflammatory gene expression (1, 6). The viral NS1 protein is composed of a N-terminal RNA-binding domain and a C-terminal effector domain. Their sequences differ among IAV strains (7, 8). NS1 strongly contributes to the virulence of the virus, and subtle nucleotide differences in the NS1 sequences may alter the pathogenic potential of a specific influenza A virus subtype (9).

Recently, several publications reported that IAV provokes massive alterations in host gene transcription (10–12). Zhao et al. (11) discovered that IAV elicits global deregulation of RNA polymerase II (RNA Pol II) transcription termination by impairing 3′-end cleavage and termination, thus ultimately resulting in global transcriptional downregulation. Bauer et al. (12) showed that host transcription is strongly altered as a result of IAV infection and found that influenza virus mounts a two-pronged attack on host transcription. First, infection leads to decreased polymerase II (Pol II) occupancy downstream of transcription start sites (TSSs), and second, interference with 3′-end processing leads to marked defects in termination of Pol II transcription at the end of genes. Similarly, Heinz et al. (10) observe global inhibition of transcription termination by NS1 causing readthrough transcription for hundreds of kilobases.

NS1-mediated host shutoff is not generally observed for all IAV strains. Particularly, strains of avian and swine origin, the mouse-adapted human laboratory strain A/PuertoRico/8/34 (PR8) and the human pandemic 2009 H1N1pdm strains, which harbor a NS1 of swine virus origin, carry NS1 proteins that do not inhibit 3′-end processing (13) due to a failure to interact with the cellular factor CPSF30 (also known as CPSF4 [cleavage and polyadenylation specificity factor 4]). The structure of the NS1 effector domain in complex with CPSF30 indicated that amino acids 184 to 188 (184-188), 103, and 106 are key amino acids for NS1-CPSF30 interaction. Indeed, NS1 (SC35M) mutated in positions 184-188 fails to interact with CPSF30 (14). The identity of these residues vary between CPSF30-blocking and -nonblocking strains. Several studies have shown that these sites are important for the process of human adaptation of different animal IAV strains (13).

It has been shown quite early on that NS1 is part of a complex that includes CPSF30 possibly in association with the IAV RNA polymerase complex (15). It was thought that the effector domain of NS1 (in this case of IAV/Udorn/72) is in complex with zinc fingers 2 and 3 of CPSF30, thus depleting CPSF30 from the CPA (cleavage and polyadenylation) complex (16). However, recent proteomics analysis of the influenza virus protein interactome has shown that NS1 from CPSF30-binding influenza virus strains is also in complex with other CPSF subunits (17). Furthermore, Bauer et al. (12) reported that the defective termination of transcription in IAV-infected host cells does not require NS1-CPSF30 interaction, questioning the relevance of the NS1-CPSF30 interaction for the dysfunction of the CPA process in infected host cells.

Finally, Anastasina et al. (18) proposed that NS1 could block the transcription of innate antiviral genes by directly binding to cellular DNA to prevent the loading of the cellular transcription machinery.

We here used a cellular model, in which the function of IAV NS1 is blocked when NS1 is fused to the mutated estrogen receptor domain ERT2 (NS1ERT) (19). We previously showed that 4-hydroxytamoxifen (OHT) induced NS1ERT from various IAV H/N subtypes as H7N7, H3N2, and H5N1 partially rescued the attenuated replication of NS1-deficient IAVs, inhibited interferon upregulation, and attenuated reporter gene expression, indicating that the addition of OHT induces a fully functional NS1 (20). Using this model, we here demonstrate that the NS1 effector domain alone—in the absence of any other viral protein—is able to trigger the suppression of host transcription and mediates global deregulation of host transcription termination. We further present evidence that nuclear speckles may represent the site of action for these NS1-mediated effects.

## RESULTS

### NS1 induces a strong attenuation of reporter gene expression driven by RNA Pol II as well as RNA Pol I promoters, but not by a phage T7 RNA promoter.

Wild-type and mutant NS1 genes fused to the modified estrogen receptor ER(T2) (NS1ERT) ER antagonist 4-hydroxytamoxifen (OHT) induce NS1 function in mammalian cells (20).

**FIG 1:**
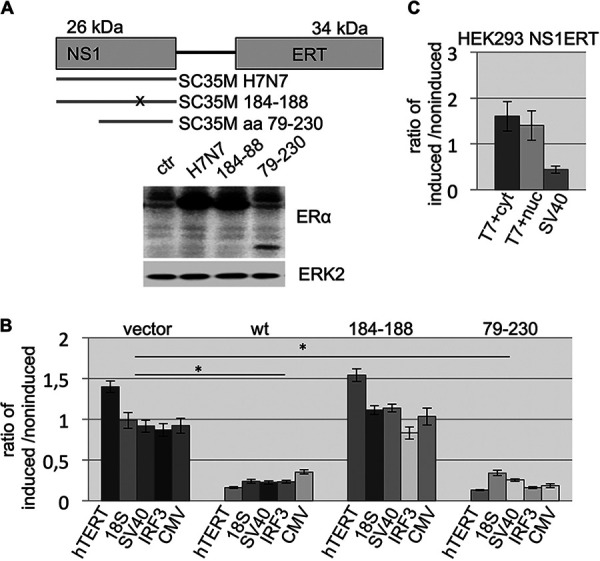
Reporter gene expression is strongly attenuated in OHT-induced cells expressing NS1ERT. (A, top) Schematic map of the constructs transduced into mammalian cells is shown expressing the fusion of wild-type NS1 and mutants and the mutated estrogen receptor domain (ERT domain) (NS1ERT). The thin line between NS1 and ERT represents a short linker consisting of amino acids PLEG. (Bottom) Western blot demonstrating the expression of the recombinant fusion proteins. SC35M, NS1 originated from IAV SC35M; SC35M 184-188, SC35M NS1 GLEWN184-188 RFKRY; SC35M aa79-230, truncated NS1 encoding only the effector domain comprising amino acid (aa) 79-230; ctr, control (empty vector-transduced cells). (B) Reporter gene assay. Reporter gene plasmids were transfected into NS1ERT-expressing cells with or without tamoxifen (OHT). The addition of OHT induces NS1 functions. Shown is the ratio of OHT-induced to noninduced cells. Vector, control cells that were transduced with an empty vector; wt, cells expressing wild-type NS1 of SC35M fused to the ERT domain; 184-188, cells expressing NS1 mutant 184-188 of SC35M fused to the ERT domain; 79-230, cells expressing wild-type truncated NS1 aa79-230 of SC35M fused to the ERT domain: hTERT, luciferase gene driven by the promoter of the human telomerase; 18S, luciferase gene driven by the upstream sequences of the ribosomal 18S RNA-encoding gene; SV40, luciferase gene driven by the promoter of the SV40 T antigen (TAg); IRF3, luciferase gene driven by the promoter of the interferon response factor 3; CMV, luciferase gene driven by the cytomegalovirus immediate early promoter. *, *P* value of <0.05 (*n* ≥ 4, average and standard deviations are shown). The significance refers for each vector to the comparison between empty vector cells versus NSERT-expressing cells or empty vector cells versus 79-230ERT, respectively. (C) HEK NS1ERT-expressing cells were cotransfected with a reporter plasmid encoding a luciferase driven by the phage T7 promoter and an expression plasmid (pAR3132 or pAR3126) that encode a T7 polymerase gene either with or without a nuclear localization signal (nuc) or without a nuclear localization signal (cytoplasmic [cyt]), respectively. As a positive control for the NS1-mediated inhibition, SV40-driven reporter plasmid (SV40) was transfected in parallel. The ratio of OHT-induced cells to noninduced cells is shown.

We first investigated whether host transcription may be affected by induction of functional NS1. Luciferase-encoding reporter gene plasmids driven by different promoters were first transfected into NS1ERT-expressing cells, which were subsequently stimulated or not with OHT. Cells that expressed wild-type NS1 and those expressing the effector domain only (NS1 amino acids [aa] 79 to 230 [79-230]) showed a strong attenuation of reporter gene transcription, irrespective of the promoter used (Fig. 1B). Interestingly, the luciferase activity driven by the human 18S rRNA promoter was also suppressed, suggesting that NS1 suppresses both RNA polymerase II and RNA polymerase I-driven expression. When the NS1 184ER antagonists 4-hydroxytamoxifen188 (GLEWN184-188 RFKRY) mutant, that is deficient in CPSF30 binding, was induced by OHT, the inhibitory effect of NS1 on RNA polymerase II and RNA polymerase I-driven transcription was completely abrogated (Fig. 1B).

We then asked whether the NS1-mediated attenuation of reporter gene expression is limited to the eukaryotic transcriptional machinery. To investigate that, a plasmid encoding the phage T7 polymerase was cotransfected with a plasmid encoding a T7 promoter-driven luciferase gene. In this scenario, induction of NS1 by OHT had no suppressive effect on luciferase activity (Fig. 1C) demonstrating that the NS1-mediated repression of reporter gene expression is indeed limited to genes driven by eukaryotic RNA polymerases.

**FIG 2:**
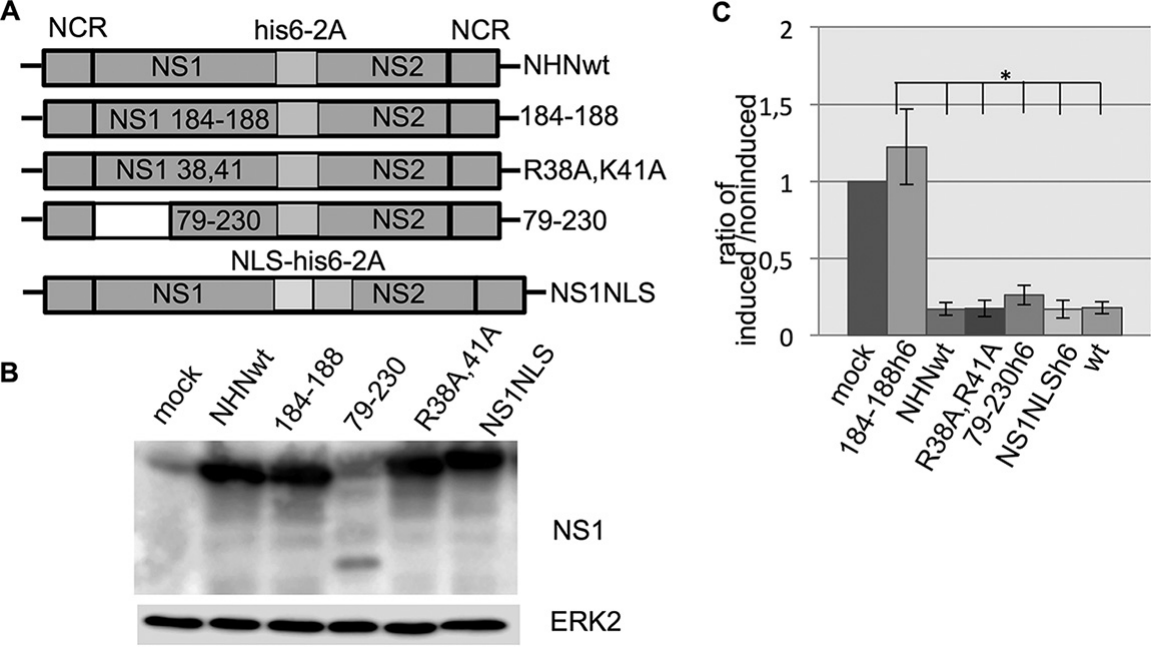
(A) A schematic map of the recombinant NS segments used in this study is shown. NS1 and NS2 are separated by a 2A peptide originating from porcine teschovirus. (B) Western blot of cells infected with recombinant IAV for 6 h. Cell lysates were harvested and blotted, and the membrane was probed with an anti-NS1 antibody. (C) Cells were infected with recombinant IAV (MOI of 5) and then immediately transfected with the reporter gene plasmid pCMV-luc. Five to 6 h after infection, cells were harvested, and the lysates were analyzed via luciferase assay. The values were normalized to the values of mock-infected cells, which were set at 1. Mock, not infected; NCR, noncoding region; his6, histidine tag; 2A, porcine teschovirus 2A peptide; NS1, nonstructural protein 1; NS2, nonstructural protein 2: NS1 184-188, recombinant IAV/segment encoding mutant NS1 GLEWN184-188 RFKRY; NS1 38,41, recombinant IAV/segment encoding mutant NS1 R38A, K41A; 79-230, recombinant IAV/segment encoding truncated NS1 aa79-230; NLS, nuclear localization signal from nucleoplasmin; NHNwt, recombinant IAV/segment encoding His-tagged wild-type NS1; NS1NLS, recombinant virus/segment encoding His-tagged wild-type NS1 fused to the nuclear localization signal.

To confirm that the observed differences in attenuation of transcription would also be detectable upon a genuine IAV infection, we generated recombinant IAVs encoding mutant NS1 proteins. To allow any desired mutation in NS1 without affecting the NS2/NEP open reading frame (orf), we designed a NS segment that contains both NS1 and NS2 orfs one after the other separated by a 2A peptide (21). We generated recombinant IAVs that encodes NS1 R38A, K41A. These mutations destroy the RNA binding ability of NS1 as well as the nuclear import signal, leading to a NS1 localization in the cytoplasm (21, 22). Furthermore, we generated a recombinant IAV that encodes the CPSF30-binding-deficient mutant NS1 184-188. Additionally, we used an IAV mutant strain encoding a truncated NS1 protein comprising amino acids 79-230 by mutating all ATG to ATC in the NS1 open reading frame until the ATG at amino acid position 79 of NS1 (NS1 79-230). Last, a recombinant IAV was generated that expresses a NS1 fused to the strong nuclear localization signal (NLS) signal from human nucleoplasmin to force NS1 into the nucleus (Fig. 2A). All NS1 variants were flagged with a His tag at their N terminus.

HEK293 cells were first infected with IAV and subsequently transfected with the reporter gene-containing plasmids. Because of the lytic nature of IAV and because the replication cycle of IAV is completed within approximately 8 h in cell culture, the incubation time after reporter gene transfection was limited for a maximal 5 to 6 h. Furthermore, for technical reasons, we investigated only reporter gene expression driven by the strong cytomegalovirus (CMV) promoter, since the luciferase activity driven by other promoters under these conditions was too weak to draw definite conclusions. Infecting cells with the NS1 mutant 184-188 IAV did not lead to suppression of luciferase activity (Fig. 2B). However, wild-type IAV as well as the IAVs either expressing the NS1 effector domain only or the RNA binding- and NLS (nuclear localization signal)-deficient (NS1 R38A, K41A) mutant were able to suppress the CMV-driven luciferase activity. Similarly, infection of cells with a recombinant virus expressing the truncated NS1 79-230 leads to a suppression of reporter gene expression. To examine whether forcing NS1 into the nucleus might enhance the suppression of reporter gene expression, we constructed an IAV encoding a NS1 with an additional strong NLS. We could not detect any additional suppressive effect on reporter gene expression by the addition of a NLS to NS1 (Fig. 2B).

In summary, the data confirm that NS1 function in OHT-induced NS1ERT-expressing cells corresponds to the function of NS1 in a genuine IAV infection process. Both the OHT-inducible cell model as well as infection experiments with recombinant IAVs demonstrate that the NS1 effector domain is sufficient to inhibit reporter gene expression. A 4-amino-acid mutation in the effector domain (NS1 mutant 184-188) abolishes the reporter gene inhibition, suggesting that the observed NS1-mediated suppression of reporter gene expression is solely a function of NS1 in a native IAV infection scenario and that no other viral protein seems to contribute to this part of the host shutoff. Furthermore, the lack of inhibition in mutant 184-188 seems to confirm the long known hypothesis that the activity of NS1 is dependent on its association with the cellular CPSF30 protein.

### NS1-mediated inhibition of reporter gene expression is not dependent on a eukaryotic polyadenylation signal.

**FIG 3:**
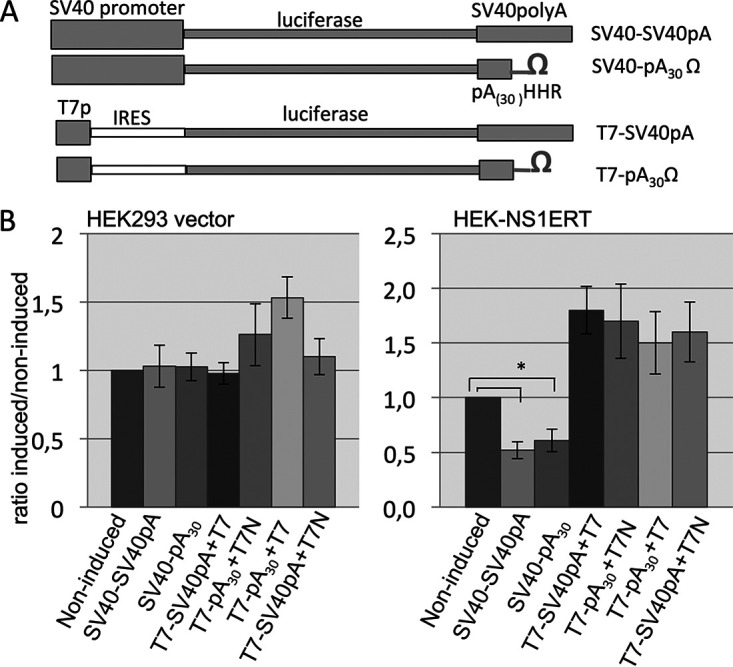
The 3′-terminal sequence of the reporter gene expression unit is not involved in the NS1-mediated inhibition of reporter gene expression. (A) A schematic map of the recombinant reporter gene plasmids is shown. (B) Empty vector control cells and NS1ERT-expressing cells were transfected with the reporter plasmids as indicated. Plasmids encoding T7 promoter-driven reporter genes were cotransfected with an expression plasmid encoding either phage T7 polymerase (T7) or phage T7 polymerase fused to nuclear localization signal (T7N). Values are shown as a ratio of OHT-induced cells to nontreated cells. SV40 promoter, SV40 early enhancer/promoter; SV40pA, SV40 late poly(A) signal; pA_(31)_, stretch of 30 deoxyadenines; Ω, self cleavage hammerhead ribozyme (HHR) sequence from hepatitis virus; SV40-SV40pA, reporter gene driven by the SV40 promoter and terminated by the SV40 polyadenylation signal; SV40pA_30_ Ω, reporter gene driven by the SV40 promoter and terminated by an artificial stretch of 30 deoxyadenines and a HHR; T7-SV40pA, reporter gene driven by the phage T7 promoter and terminated by the SV40 polyadenylation signal; T7-pA_30_Ω, reporter gene driven by the phage T7 promoter and terminated by an artificial stretch of 30 deoxyadenines and a HHR; T7p, T7 promoter; IRES, internal ribosome entry site from EMCV (encephalomyocarditis virus).*, *P* value <0.05 (*n* ≥ 4; averages ± standard deviations [error bars] are shown).

Since the only NS1 mutant that does not suppress reporter gene activity is the NS1 184-188 that fails to bind to CPSF30, we assumed that CPSF30 may be involved in the observed suppression of reporter gene expression. CPSF30 plays a key role in pre-mRNA 3′-end formation, recognizing the polyadenylation (polyA) signal and interacting with polyA polymerase and other factors to confer cleavage and polyA addition. All reporter gene constructs described above encode a eukaryotic simian virus 40 (SV40) polyadenylation signal at the 3′ termini of the luciferase expression cassette except the T7 RNA polymerase-driven pT7T7/T7luc vector (23). Thus, the question arose whether the interaction of NS1 with CPSF30 may lead to a dysfunctional 3′ termination of transcription and thus to a reduction in reporter gene expression. Therefore, we replaced the SV40 polyA signal with a nonfunctional, synthetic stretch of 30 deoxyadenines followed by a ribozyme sequence (Fig. 3A) and transfected these constructs into HEK293 vector control as well as HEK-NS1ERT cells. Comparison between OHT-induced and noninduced cells revealed that OHT induction of NS1 in cells transfected with the SV40 promoter-driven constructs leads to a reduction of reporter gene activity irrespective of the 3′ terminus (Fig. 3B). Furthermore, no OHT-induced inhibition of reporter gene activity could be observed when the T7 RNA polymerase-driven reporter constructs carrying either a eukaryotic SV40 polyA signal or a nonfunctional oligo(dA) stretch were cotransfected with a plasmid encoding either a nuclear localized T7 RNA polymerase or a cytosolically active T7 RNA polymerase into NS1ERT cells. Thus, the transfer of a eukaryotic polyadenylation signal to the 3′ termini of a T7-driven expression cassette did not confer the sensitivity for suppression of its expression by NS1. These data suggest that the NS1-induced inhibition of reporter gene expression is not dependent on a functional eukaryotic polyadenylation signal, which is the site where CPSF30 is thought to act. Instead, a eukaryotic promoter driving a eukaryotic transcription machinery seems to be essential for the ability of NS1 to suppress the reporter gene activity.

### Transcription is attenuated in OHT-induced NS1ERT-expressing cells.

**FIG 4:**
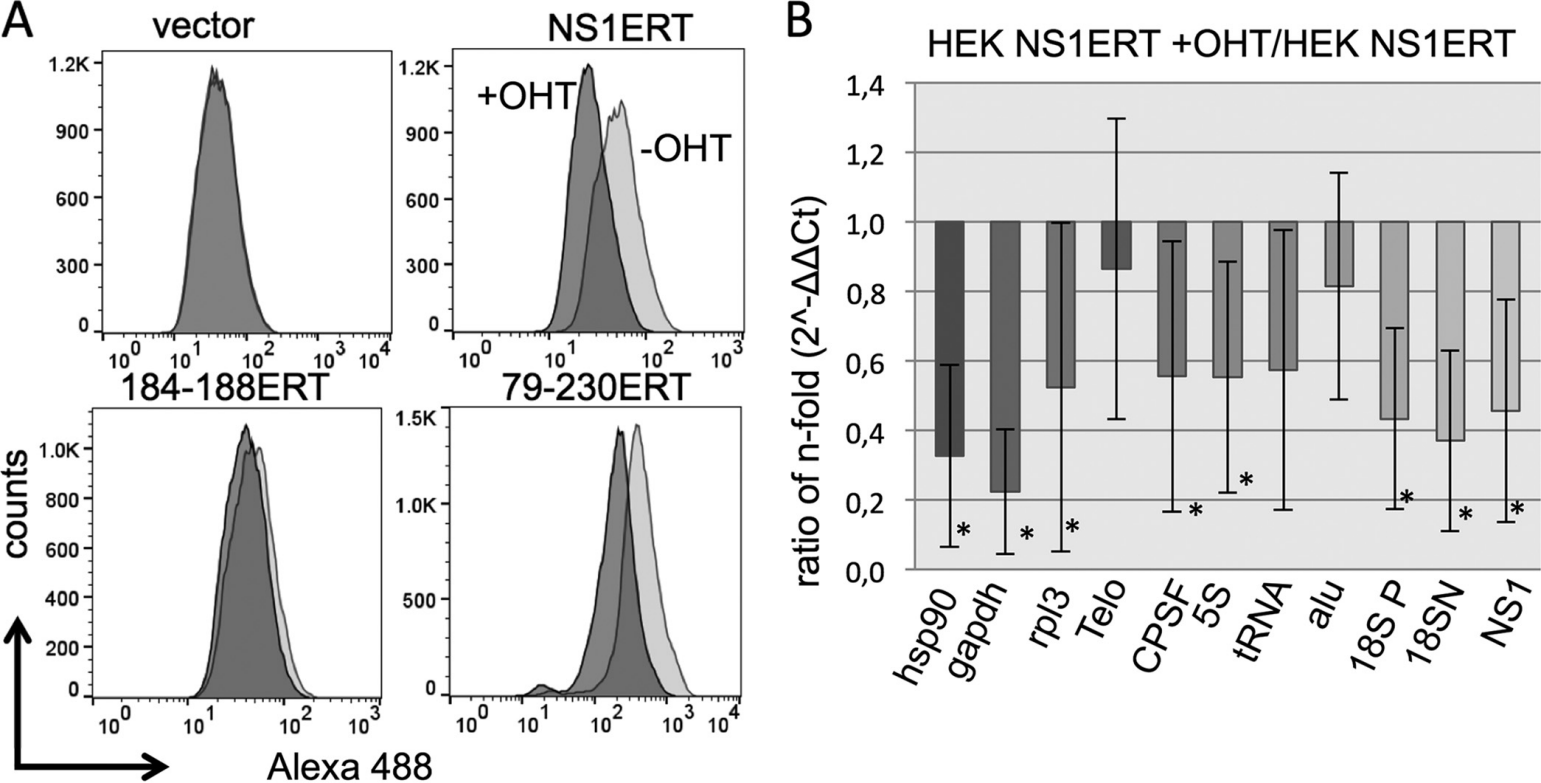
Global transcription is inhibited upon induction of a functional NS1 upon OHT addition. (A) OHT was added to NS1ERT-expressing cells to induce a functional NS1. One hour after OHT induction, ethynyl uridine (EU) (1 mM) was added to the medium for 2 h. Subsequently, cells were fixed, and a fluorophore (Alexa Fluor 488) was added by click chemistry and finally analyzed by FACS. Dark gray shading indicates treatment with OHT; light gray shading indicates treatment without OHT. Vector, control cells transduced with an empty vector; NSERT, NSERT-expressing cells; 184-188ERT, mutant NS1-184-188ERT-expressing cells; 79-230ERT, truncated NS1 79-230ERT-expressing cells. (B) Cells were first transfected with *in vitro*-generated ethynyl-uridine-labeled RNA encoding luciferase. Subsequently, cells were seeded into wells and treated without OHT. After OHT addition, cells were incubated with ethynyl-uridine (EU) for 2 h, RNA was isolated and biotinylated, and streptavidine-isolated RNA was analyzed by qRT-PCR. The threshold cycle (Ct) values were normalized to those of luciferase cDNA, and the 2^−ΔΔCt^ values were calculated. Finally, the values of the OHT-induced cells were compared to those of the noninduced cells (ratio). hsp90, heat shock protein 90; gapdh, glyceraldehyde-3-phosphate dehydrogenase; rpl3, ribosomal protein L3; Telo, telomerase; CPSF, cleavage and polyyadenylation specificity factor; 5S, 5S RNA; tRNA, tRNA tryptophan; alu, genomic alu repeat; 18S P, 18S RNA; 18SN, 18S RNA (see Table S1 in the supplemental material for primer sequences); NS1, NS1 from IAV SC35M encoded in NS1ERT. *, *P* value of <0.05 (*n* ≥ 8; averages ± standard deviations [error bars] are shown). The significance refers to the ratio of the expression level between noninduced and OHT-induced cells. The noninduced expression level of the genes was set at 1.

The NS1-mediated suppression of reporter gene activity could be due to a number of possible molecular mechanisms. To investigate whether the host gene transcription rate is attenuated upon NS1 induction, NS1ERT-expressing cells were stimulated with OHT or solvent. After 1 h of stimulation, ethynyl-uridine (1 mM) was added to the cells for 30 min to be incorporated into nascently transcribed RNA. Subsequently, the cells were fixed and permeabilized, and a fluorophore was chemically bound to nascently transcribed ethynyl-labeled RNA. The cells were then analyzed by flow cytometry (fluorescence-activated cell sorting [FACS]). The fluorescence intensity was reduced in OHT-induced NS1ERT- and in NS1 79-230ERT-expressing cells versus noninduced cells, but not so in NS1 184-188ERT-expressing cells (Fig. 4A). This experiment demonstrated that global transcription is suppressed by wild-type NS1 as well as by the NS1 effector domain. Again, mutation of the CPSF30 binding site aa 184-188 in NS1 reverted the transcriptional inhibition.

10.1128/mBio.02196-21.4 TABLE S1 Sequences of the primers used for RT-PCR. Download Table S1, DOCX file, 0.08 MB. Copyright © 2021 Nacken et al. 2021 Nacken et al. https://creativecommons.org/licenses/by/4.0/ This content is distributed under the terms of the Creative Commons Attribution 4.0 International license.

We then wished to confirm the NS1-mediated suppression of host gene transcription, which we observed by FACS analysis also by qRT-PCR (quantitative reverse transcriptase PCR). *In vitro* ethynyl-UTP-labeled RNA encoding luciferase was first transfected into NS1ERT-expressing cells. Subsequently, equal cell numbers were seeded and stimulated with or without OHT. Both cell populations were then treated with ethynyl-uridine to label nascently transcribed RNA. Total RNA was purified from the cells and chemically labeled with biotin via click chemistry. Finally, the biotinylated RNA was streptavidin isolated and reverse transcribed and used for qRT-PCR. The transfected, *in vitro*-generated luciferase-encoding RNA was used as an “internal” reference for evaluation.

Five RNA polymerase II-driven genes were analyzed: HSP90AA1 (hsp90), GAPDH (glyceraldehyde-3-phosphate dehydrogenase), RPL3 (ribosomal protein L3), TEP1 (telomerase), and CPSF30. The expression of all of them except the telomerase mRNA were significantly reduced upon OHT treatment. Also, the amount of the RNA polymerase III-driven 5S RNA and tRNA as well as the RNA polymerase I-driven 18S RNA was significantly reduced. The transcription of alu sequences that are transcribed by RNA Pol III was not significantly diminished (Fig. 4B).

### CPSF30 cotransfection counteracts the NS1-mediated repression of RNA Pol II-driven reporter gene expression.

**FIG 5:**
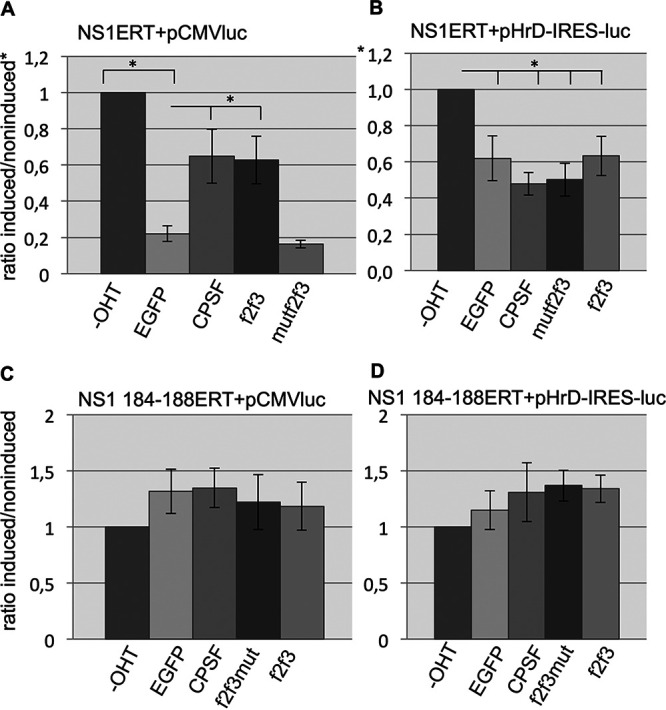
Overexpression of CPSF30 counteracts the NS1-mediated inhibition of RNA Pol II promoter-driven reporter gene (pCMV-luc) (A), but not of an RNA Pol I promoter-driven reporter gene (pHrD-IRES-luc) (B). NS1ERT-expressing cells were cotransfected with expression vectors encoding enhanced green fluorescent protein (EGFP) as a control, CPSF30, the NS1 binding zinc finger domains f2f3 of CPSF30, and a mutant, nonactive f2f3 (mutated zinc fingers) of CPSF30. Cells were treated without OHT (-OHT), and the ratio of the relative light units (RLU) of OHT-treated versus nontreated cells is shown. No inhibition of reporter gene expression is observed using cells expressing mutant NS1 184-188 (C and D).

We further investigated whether overexpression of CPSF30 can overcome the NS1-mediated transcriptional inhibition (see Fig. S1 in the supplemental material). Cotransfection of CPSF30 into OHT-treated, NSERT-expressing cells partially eliminated the NS1-mediated inhibitory effect on reporter gene expression. Similarly, transfection of the zinc finger domains f2f3 of CPSF30, which is known to bind NS1 (24), led to an attenuation of inhibition. However, cotransfection of a f2f3 domain of CPSF, whose zinc finger motifs have been mutated, thus being unable to bind NS1, failed to weaken the NS1-mediated inhibitory effect (Fig. 5A). Cotransfection of CPSF30 had no effect on the NS1-mediated inhibitory effect on the expression of the reporter gene controlled by a RNA polymerase I promoter (Fig. 5B). As expected, CPSF30 cotransfection does not change the reporter gene activity using cells that express the noninhibiting NS1ERT mutant 184-188 (Fig. 5C and D).

10.1128/mBio.02196-21.1 FIG S1 Control of expression of recombinant CPSF30 protein and deletion mutants. Cells were transfected with pcDNA3 expression vectors encoding 1xmyc-CPSF4, 5xmyc-f2f3 (CPSF30), 5xmyc-f2f3 mutant (CPSF30), and EGFP (see Fig. 5). Protein lysates were analyzed by Western blotting. The membrane was probed with an anti-myc antibody or with an anti-CPSF30 antibody, respectively. Probing the membrane with anti-erk2 antibody (SantaCruz, USA) served as a loading control. Download FIG S1, TIF file, 0.4 MB. Copyright © 2021 Nacken et al. 2021 Nacken et al. https://creativecommons.org/licenses/by/4.0/ This content is distributed under the terms of the Creative Commons Attribution 4.0 International license.

Altogether, these data suggest that the NS1-mediated inhibition of RNA polymerase II-driven host gene transcription may be caused by the deprivation of CPSF30 from the transcriptional machinery.

### siRNA-mediated knockdown of endogenous CPSF30 has no effect on reporter gene expression.

**FIG 6:**
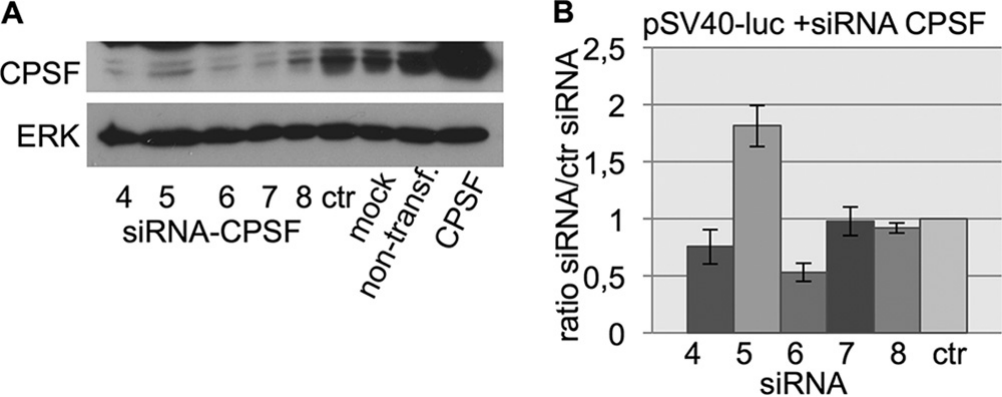
CPSF30 knockdown does not inhibit reporter gene transcription. (A) Western blot showing the efficiency of the CPSF30 siRNA knockdown 48 h after siRNA transfection. siRNA-CPSF 4 to 8 indicate different siRNAs from different suppliers (see supplemental material). ctr, nonspecific control siRNA as a negative control; non-transf., nontreated cells; CPSF, cells transfected with a plasmid expressing CPSF30 as a positive control; ERK, ERK2 antibody was used as a loading control. (B) Reporter gene assay. Cells were transfected with siRNAs as indicated (siRNA sequence see Table S2 in the supplemental material). After 24 h, cells were transfected with the reporter gene plasmid. The next day, the cells were harvested and analyzed.

Assuming that the cellular availability of CPSF30 limits reporter gene expression, knockdown of the endogenous gene should result in reduced levels of the luciferase activity. Thus, a small interfering RNA (siRNA)-induced knockdown of CPSF30 is assumed to similarly deprive CPSF30 from the transcriptional machinery as NS1 is supposed to do. However, using five different siRNAs, we did not observe a reliable decrease of reporter gene expression compared to control siRNA-treated cells (Fig. 6A and B). This finding may be due to the possibility that residual CPSF30 is sufficient to maintain the level of reporter gene expression or questions the hypothesis that the cellular availability of CPSF30 controls the reporter gene activity.

10.1128/mBio.02196-21.5 TABLE S2 List of CPSF30 siRNAs used in this study. The asterisks indicate sequences that were extracted from W. Chen et al., 2013, PLoS One (doi:https://doi.org/10.1371/journal.pone.0082728). Download Table S2, DOCX file, 0.06 MB. Copyright © 2021 Nacken et al. 2021 Nacken et al. https://creativecommons.org/licenses/by/4.0/ This content is distributed under the terms of the Creative Commons Attribution 4.0 International license.

### A functional NS1 effector domain is also essential for the defect in 3′ termination of host transcription.

IAV infection provokes a global deregulation of RNA Pol II transcription termination by impairing 3′-end cleavage and termination (10–12). We here infected cells with wild-type IAV (SC35M) and mutant IAV NS1 184-188 for 6 h and then investigated the host cell transcriptome by transcriptome sequencing (Ranse) analysis.

**FIG 7:**
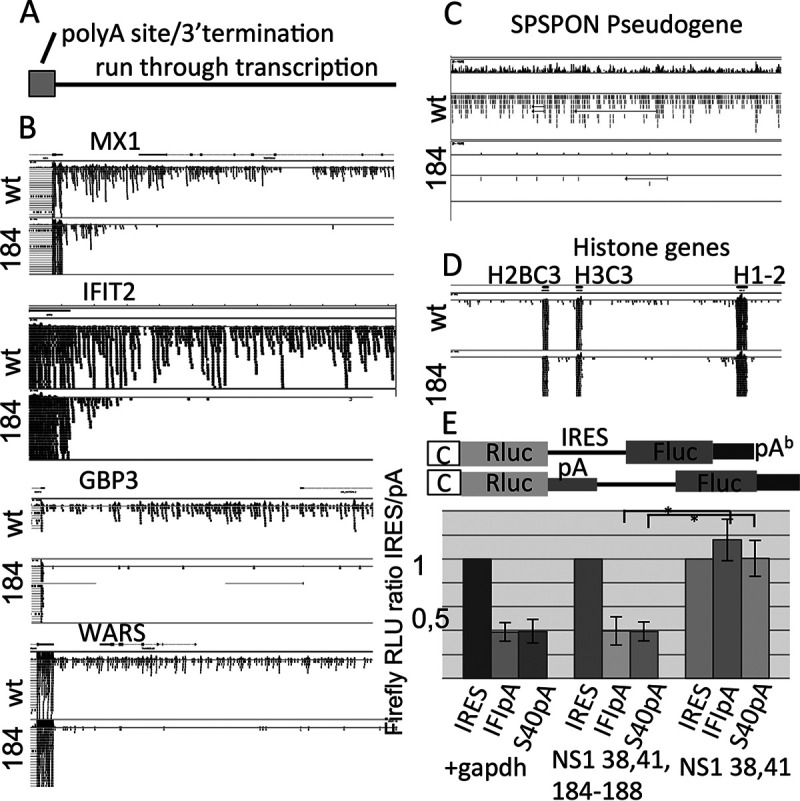
The IAV-induced deregulation of 3′ termination of host transcription is not observed in cells infected with IAV NS1 184-188. (A to D) Analysis of a RNASeq experiment. The cells were infected with wild-type (wt) IAV or IAV NS1 184-188. The Integrative Genome Viewer software was used to align the reads along the human genome hg38. (A) Schematic representation of the regions of the genes that are aligned in panel B. (B) The 3′-terminal region of four genes (MX1, FIT2, GBP3, and WARS) is schematically shown, including the reads that align downstream of the 3′ terminus of transcription. The height of the bars or number of dots represent the numbers of reads homologous to the specific site of the sequence. wt, downstream transcription in cells infected with wild-type IAV; 184, downstream transcription in cells infected with IAV NS1 184-188. (C) Alignment of reads in a region encoding the pseudogene SPSON that is not transcribed or is rarely transcribed in humans. (D) Alignment of reads in a region encoding histone genes. (E) Cotransfection experiment. (Top) Schematic representation of the bicistronic expression cassette with and without a polyadenylation signal 5′ of the internal ribosomal entry site (IRES) used to quantitate the extent of readthrough transcription via a reporter gene assay. Rluc, renilla luciferase; C, CMV promoter; IRES, internal ribosomal entry site; Fluc, firefly luciferase; pA, polyadenylation signal from IFI44 or SV40TAg; pA^b^, bovine growth hormone polyadenylation signal. (Bottom) The reporter gene plasmids were cotransfected along with a control plasmid (gapdh) or a NS1-encoding plasmid (NS1 38,41 or NS1 38,41,184-188). The RLU of firefly luciferase activity was related to that of the control (gapdh) (was set at 1). *, *P* value of<0.05 (*n* ≥ 4; averages ± standard deviations are shown). IRES, bicistronic cassette containing plasmid and both orfs are separated by an IRES element; IFIpA, bicistronic cassette containing plasmid containing the polyadenylation signal of the IFI44 gene 5′ of the IRES element; SV40pA, bicistronic cassette containing plasmid containing the polyadenylation signal of the SV40 TAg gene 5′ of the IRES element.

The alignment of the reads to the human model genome confirms the deregulation of RNA Pol II transcription termination upon wild-type IAV infection. However, comparing transcriptional termination in wild-type IAV-infected cells and mutant NS1 184-188-infected cells revealed that no deregulation of 3′ termination of host transcription could be observed in cells infected with the mutant IAV NS1 184-188 (Fig. 7), confirming that the effector domain of NS1 is also responsible and sufficient for the deregulation of the 3′ termination of host cell transcription. Additionally, usually nontranscribed regions of the genome as pseudogenes are transcribed in cells infected with wild-type IAV but not when infected with IAV NS1 184-188 (Fig. 7C). The deregulation does not uniformly affect all genes. As seen in Fig. 7D, the transcriptional termination of the histone genes is hardly altered. These findings are largely in agreement with recent reports (10–12).

To further confirm these data, a bicistronic reporter gene expression cassette was constructed, in which both open reading frames are separated by an internal ribosome entry site (IRES) sequence. Between the 5′ located open reading frame and the IRES sequence, a polyadenylation signal sequence was cloned. This signal terminates RNA Pol II transcription and thereby blocks the expression of the downstream reporter gene. Cotransfection of this bicistronic reporter gene plasmid along with NS1 expression plasmids allowed the quantification of the dysregulation of the RNA Pol II transcriptional termination, since the expression of downstream reporter gene will be related to the efficiency of the polyadenylation site. Indeed, we observed that the cotransfection of NS1 38,41 results in significant higher readthrough transcription and by that higher luciferase activity than the cotransfection with the mutant NS1 184-188 (Fig. 7E), suggesting that the RNA Pol II in the presence of NS1 largely ignores the polyadenylation signal, runs through this termination signal, and transcribes the downstream reporter gene. We do not observe a difference between the two polyadenylation signals used in our experiments, suggesting that the run through transcription induced by NS1 is most likely not dependent on the polyadenylation signals of specific genes. The data of the reporter gene assay greatly reflect the data obtained by the RNASeq experiments.

### Immuncytochemical analysis via STORM microscopy rises the question whether NS1-mediated transcriptional inhibition of reporter gene expression is dependent on nuclear localization of NS1.

**FIG 8:**
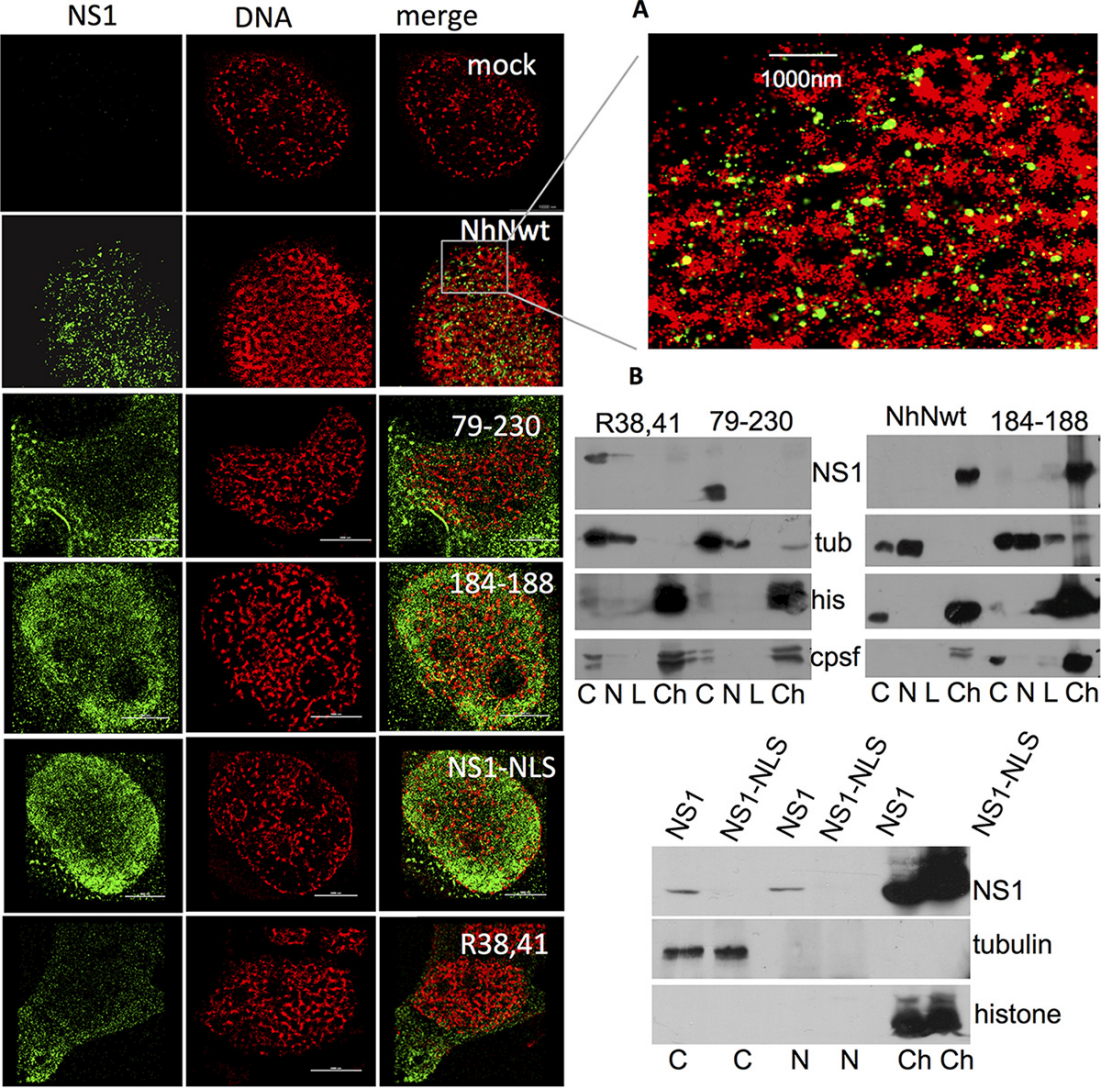
STORM microscopy and biochemical fractionation suggest that chromatin association of NS1 is not essential for inhibiting host gene expression. (A) Cells were treated overnight with a low concentration (1 μM) of EdU (5-ethynyl-2′-deoxyuridine). The next day, they were infected with recombinant IAV as indicated. Five hours after infection, cells were fixed, and a fluorophore was added to the ethynyl moiety by click chemistry to label cellular DNA. The fixed cells were then immunostained with an anti-His-tagged antibody and applied to STORM microscopy. mock, not infected; NS1, antibody against NS1; 184-188, recombinant IAV encoding mutant NS1 GLEWN184-188 RFKRY; 38,41, recombinant IAV encoding mutant NS1 R38A, K41A; 79-230, recombinant IAV encoding truncated NS1 aa79-230; Nh6Nwt, recombinant IAV encoding His-tagged wild-type NS1; NS1NLSh6, recombinant virus/segment encoding His-tagged wild-type NS1 fused to the nuclear localization signal (NLS). (B) Western blot of samples of the biochemical fractionation of infected cells. NS1, antibody against NS1; tubulin, antibody against tubulin; histone, antibody against histone; C, cytosol fraction; N, nuclear fraction; L, low salt chromatin fraction; Ch, chromatin fraction after low salt extraction.

The host transcriptional machinery, including CPSF30, is located in the nucleus. NS1 is known to be located in the nucleus as well as in the cytoplasm. To investigate whether NS1-mediated transcriptional inhibition correlates with nuclear localization of NS1, cells were infected with recombinant wild-type and mutant IAVs (Fig. 1A). We analyzed the cellular localization of NS1 by superresolution stochastic optical reconstruction microscopy (STORM) microscopy and by biochemical fractionation of infected cells. Wild-type NS1 as well as mutant NS1 184-188 was mainly found in the chromatin fraction of the nucleus. However, both the mutant NS1 R38A, K41A (NS1 38,41) and the truncated NS1 79-230 were located in the cytosol (Fig. 8). Of note, via biochemical fractionation, we did not detect NS1 38, 41 or NS1 79-230 in the nucleus (Fig. 8B). On the other hand, the NS1-NLS mutant seems to be exclusively bound to the chromatin fraction of the nucleus.

STORM microscopy revealed that only a small fraction of NS1 actually colocalized with genomic DNA (Fig. 8A). The finding that both NS1 mutant 38, 41 and the truncated NS1 79-230 according to this experiment are located in the cytosol are potent suppressors of transcriptional inhibition (Fig. 2B) questioned the assumption that nuclear localization of NS1 is essential for the inhibition of reporter gene expression.

### A disturbance of the balance between nuclear and cytosolic localized NS1 does not attenuate inhibition of reporter gene expression.

**FIG 9:**
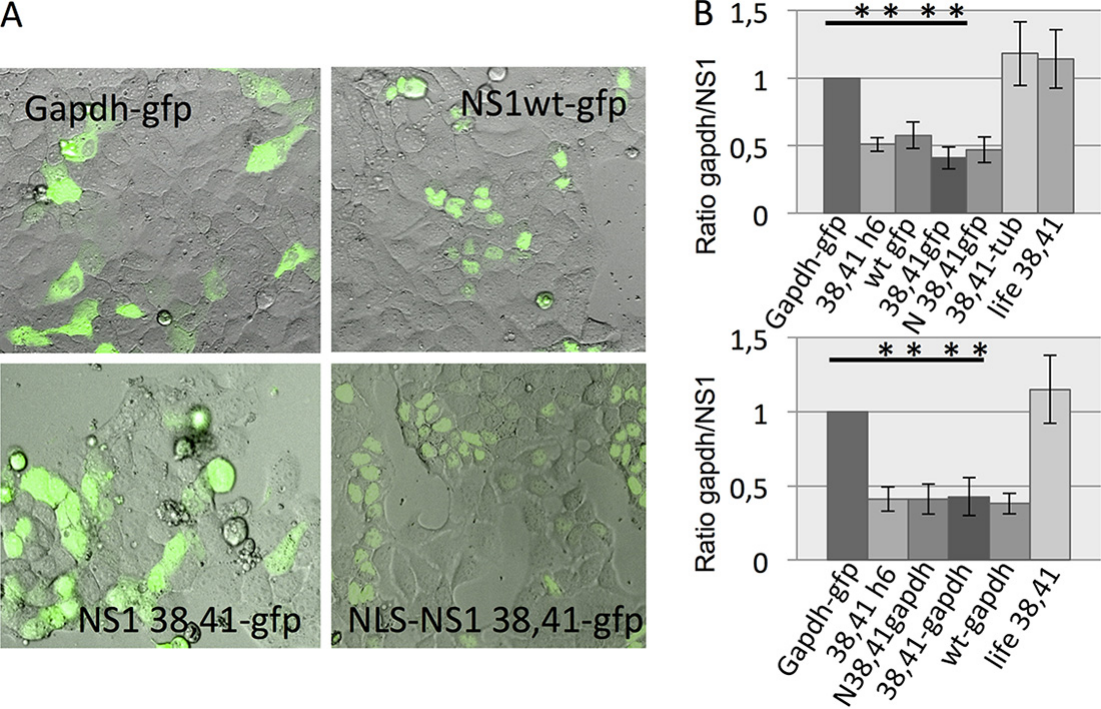
Alteration of the subcellular distribution of NS1 does not affect the NS1-mediated host gene shutoff. (Left) Cells were transfected with the pcDNA3 expression vector encoding gfp fusion proteins either fused to gapdh as a control or wild-type NS1 (NS1wt-gfp), mutant NS1 R38A, K41A (NS1 38,41-gfp), or NS1 R38A, K41A containing a nuclear localization signal at the N terminus (N-NS1 38,41-gfp). (Right) Reporter gene assays are shown. Cells were cotransfected with a reporter gene construct and a NS1-encoding expression vector. Luciferase activity of gapdh coexpressed luciferase was set at 1. Gapdh-gfp, gapdh fused to gfp (as a cytosolic control); 38,41h6, NS1 R38A, K41A with His tag; wtgfp, wild-type NS1 fused to gfp; 38,41gfp, NS1 R38A, K41A fused to gfp; N 38,41gfp, NS1 R38A, K41A fused to gfp containing a N-terminal nuclear localization signal; tub-38,41, fusion between NS1 R38A, K41A and alpha tubulin; life 38,41, NS1 R38A, K41A N-terminally fused to an actin binding lifeAct sequence. Also seen are the corresponding NS1 fusions with gapdh. *, *P* value <0.05 (*n* ≥ 4; averages ± standard deviations are shown). Significance as indicated by the asterisks refers in each case to the comparison of gapdh-gfp-expressing cells (set at 1) with the cells expressing modified NS1-encoding protein.

To investigate the question of whether nuclear localization is dispensable for NS1-mediated host shutoff, we constructed NS1 fusion proteins that force NS1 to subcellular sites other than the nucleus. To be able to observe the localization of NS1 in living cells, we fused NS1 to gfp (green fluorescent protein) and repeated these experiments. NS1 wild-type-gfp, NS1 38, 41-gfp as well as NLS-NS1-38, 41-gfp inhibited reporter gene expression to a similar level. However, they differ in their subcellular distribution (Fig. 9A). Fusing NS1 to the cytoskeletal protein actin or tubulin abrogates its inhibitory effect on reporter gene expression most likely because these NS1 fusions are either excluded from the nucleus or at least spatially confined to filaments. However, a fusion of wild-type N3 and mutant NS1 38, 41 to the cytosolic GAPDH did not impair inhibitory function (Fig. 9B). A NS1 38, 41-GAPDH fusion containing a nuclear localization signal (NLS) inhibited reporter gene expression similar to expression of the NS1 versions without NLS. These data suggest that a shift in the balance from nuclear to cytosolic NS1 does not affect the shutoff of gene expression. We further used the split-gfp system to visualize NS1 fusion proteins and to minimize potential steric effects on NS1 by fusing full-length gfp. The gfp 11 domain was N-termially fused to NS1 38,41-tubulin and NS1 38,41-lifeAct and cotransfected with reporter gene constructs into cells stably tranduced with pQCXIP-gfp1-10 (Addgene no. 68715) (Fig. S2). These data indicate that NS1 when relocated to the cytoskeleton of the cell is no longer able to suppress reporter gene expression (Fig. 9B and Fig. S3). Taken together, the results could not confirm the data obtained by STORM that mutant NS1 38, 41 is exclusively present in the cytosol. Instead, at least the presence of a small proportion of the NS1 molecules in the nucleus seems to be necessary for host gene shutoff.

**FIG 10:**
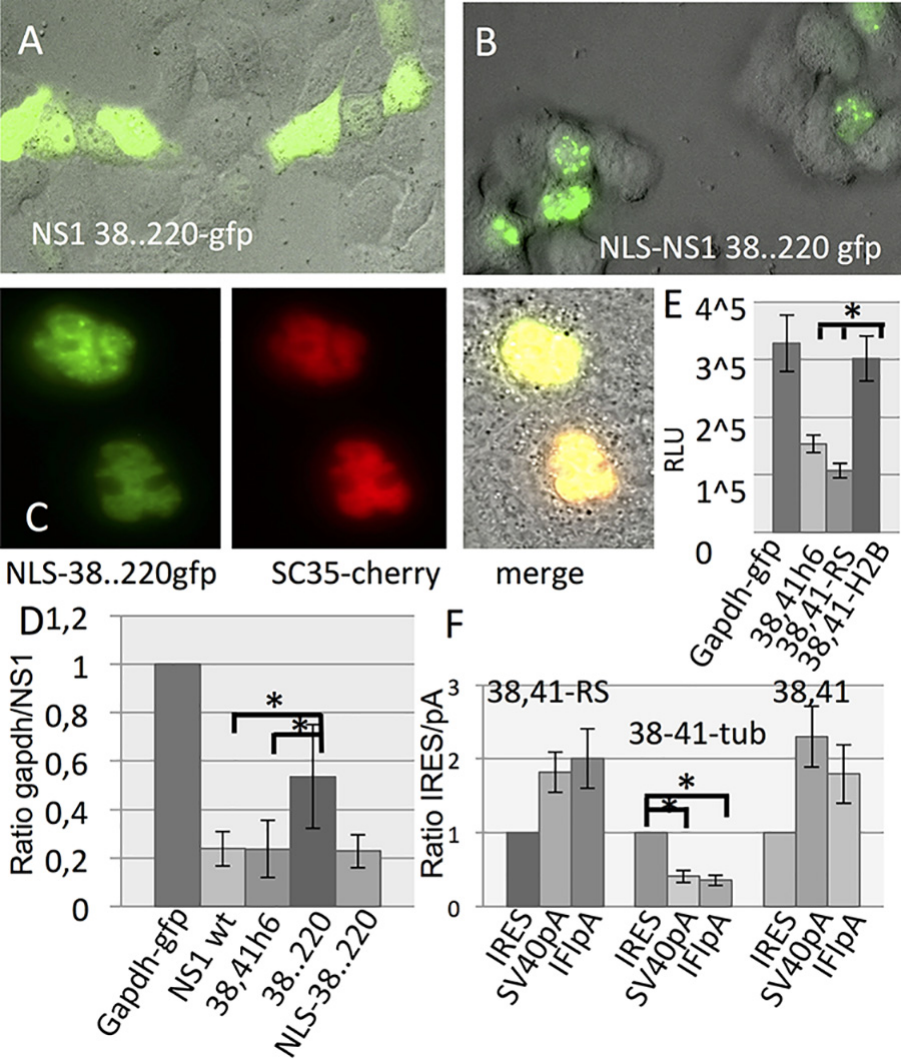
The presence of NS1 in granular structures of the nucleus correlates with inhibition of host gene expression. The subcellular distribution of GFP fusions of the quadruple mutant NS 38, 41, 219, 220 (R38A, K41A, K219A, R220E) which is deficient of both nuclear localization sites (NLS) without (A) and with (B) a N-terminally located NLS of SV40 TAg. Expression plasmid was transfected into cells and analyzed miicroscopically. (C) Colocalization experiment. Cells cotransfected with NLS-NS 38, 41, 219, 220-gfp and SC35-mcherry-expressing plasmids were microscopically analyzed. (D) Cells were cotransfected with reporter gene construct (pSV40-luc) and the indicated NS1-encoding expression vector. The resulting luciferase activity is shown as a ratio of the control (gapdh cotransfection, set at 1). (E). NS1 fusion proteins were cotransfected with reporter gene plasmid. The data are shown in relative light units (RLU). (F) Cells were cotransfected with the bicistronic constructs (schematic representation of reporter gene plasmids, see Fig. 7e) and a modified NS1-encoding plasmid as indicated. The resulting luciferase activity is shown as a ratio of control/NS1 (IRES cotransfection, set at 1). 39,41h6, NS1 R38A, K41A h6 (histidine tag); 38..220, mutant NS1 R38A, K41A, K219A, R220E; NLS, nuclear localization site of SV40 TAg; 38,41-RS, NS1 R38A, K41A h6 fused to the RS (arginine-serine-rich) domain of nuclear marker protein SC35; 38,41-H2B, NS1 39, 41h6 fused to histone 2B; 38,41-tub, NS1 R38A, K41A h6 fused to tubulin; IRES, plasmid encoding bicistronic renilla luciferase and firefly luciferase, separated by an IRES sequence; SV40pA, bicistrinic plasmid containing an SV40 polyadenylation site 5′ of IRES; IFIpA, biscistronic plasmid containing the polyA site of IFI44 gene 5′ of IRES (for schematic representation of plasmids, see Fig. 7e).

10.1128/mBio.02196-21.2 FIG S2 Control of expression of recombinant NS1 and NS1 fusion proteins. Cells were transfected with pcDNA3 expression vectors encoding the quadruple mutant NS1 R38A, K41A, K219A, R220E, the NS1 R38A, K41A h6 (his tagged) fused to the RS (arginine-serine-rich) domain of nuclear marker protein SC35, NS1 R38A, K41A h6 fused to H2B, NS1 R38A, K41A h6 N-terminally fused to an actin binding lifeAct sequence, marker protein SC35, NS1 R38A, K41Ah6 fused to H2B, NS1 R38A, K41Ah6 fused to alpha tubulin and NS1 R38A, K41Ah6 fused to gapdh (see Fig. 9 and 10). Protein lysates were analyzed by Western blotting. The membrane was probed with an anti-NS1 antibody (geneTex, USA). Probing the membrane with anti-erk2 antibody (SantaCruz, USA) served as a loading control. Download FIG S2, TIF file, 0.4 MB. Copyright © 2021 Nacken et al. 2021 Nacken et al. https://creativecommons.org/licenses/by/4.0/ This content is distributed under the terms of the Creative Commons Attribution 4.0 International license.

10.1128/mBio.02196-21.3 FIG S3 NS1-tubulin fusion deficient in mediating host shutoff is excluded from the nucleus. To demonstrate that the NS1-tubulin fusion protein is *in vivo* located at nonnative sites compared to wild-type NS2, we used a gfp protein complementation assay (PCA). This assay allows the detection of the protein of choice without gross structural alteration like a full gfp fusion. A nucleotide sequence encoding the peptide MEKRDHMVLHEYVNAAGIT that encodes the 11th domain of sfGFP (superfolder GFP) (followed by a GGGS linker region that was fused to the NS1 38,41-alpha tubulin fusion protein (gfp11-NS1 38,41-tubulin). Since it is known that the 11th domain of sfGFP self-assembles with a sfGFP containing domain 1-10, the expression vector encoding GFP11-NS1 38, 41-tubulin was transfected into cells that had been retrovirally transduced with pQCXIP-GFP1-10 (Addgene no. 68715). For a control, a GFP11-NS1 (wild-type NS1) (gfp11-NS1 wt)-encoding vector was transfected. Download FIG S3, TIF file, 0.5 MB. Copyright © 2021 Nacken et al. 2021 Nacken et al. https://creativecommons.org/licenses/by/4.0/ This content is distributed under the terms of the Creative Commons Attribution 4.0 International license.

### The presence of NS1 in granular structures of the nucleus correlates with inhibition of host gene expression.

The basic amino acid position K219 and R220 of NS1 are highly conserved and are part of a putative second NLS of NS1 (24–26). Thus, we asked whether destruction of both NLS would reduce or even abolish nuclear localization of NS1 and how this would influence inhibition of reporter gene expression. The quadruple mutant NS1 38, 41, 219, 220 (R38A, K41A, K219A, R220E)-gfp fusion protein is localized to granular structures within the nucleus (Fig. 10A and B). This mutant was not able to inhibit reporter gene expression as strongly as NS1 38,41 (Fig. 10D). However, relocating this mutant to the nucleus by fusing a SV40 tag nuclear localization signal (PKKKRR) to the N terminus restores the ability to suppress luciferase activity, suggesting that the addition of the synthetic NLS to the mutant NS1 38, 41, 219, 220 guides NS1 to its native site in the nucleus (Fig. 10D and Fig. S3). Furthermore, the quadruple mutant NS1-gfp colocalizes with the nuclear speckle marker protein SC35 fused to mcherry (Fig. 10C). Finally, cotransfection experiments with NS1 38, 41 fused to the RS domain of the nuclear speckle marker protein SC35, known to be necessary and sufficient to guide SC35 to speckles (27), inhibits reporter gene expression similar to nonfused NS1 38,41. In contrast, cotransfection with NS1 38,41 fused to H2B (histone 2B) failed to attenuate reporter gene expression (Fig. 10E and Fig. S3). In addition, cotransfection of the bicistronic reporter gene plasmids used to analyze transcription termination with the NS1 38,41-RS indicates that NS1 38,41-RS and NS1 38,41 induce the RNA Pol II to read through the SV40 or IFI44 polyadenylation sites, whereas a NS1 38,41 fused to tubulin is not able to trigger RNA Pol II to ignore the polyadenylation sites. These data suggest that nuclear speckles are most likely the sites of action where the multifunctional NS1 needs to be present to comply the inhibition and deregulation of host gene transcription.

## DISCUSSION

Multiple mechanisms of host shutoff by influenza A virus have been described. This process involves at least six viral proteins: The trimeric RNA-directed RNA polymerase complex (RdRp) consisting of PB1, PB2, and PA, the nonstructural protein NS1, and PA-X (28). It has been shown earlier that IAV-infected cells display a reduced level of both the synthesis and half-life of host mRNAs but also a reduction in host protein translation (28). However, to date, no study has tested the full extent of the contribution of NS1 on host gene expression. This mainly relies on toxic cell effects due to the constitutive expression of NS1 (29–31). We could overcome this problem by generating an inducible version of NS1 that enables us to study biological functions of NS1 in the absence of toxic effects (20).

We show here that NS1 inhibits host gene transcription without the need of any other viral proteins or other viral functions, thereby identifying NS1 a major player in mediating the host gene shutoff. We further demonstrate that the functional effector domain of NS1 (aa 79-230) is sufficient for this function. Reporter gene assays, FACS analysis, and qRT-PCR experiments demonstrate that the transcriptional inhibition of host genes is not restricted to RNA polymerase II-driven genes. On the other hand, the qRT-PCR data also indicate that there may be individual genes or expression units that are either unresponsive or do not respond to the same extent to the action of NS1 as others. A recent report (32) confirms that the virus reduces the amount of mRNA in the host cells to take over the mRNA pool. In line with these findings, our analysis indicates that a eukaryotic RNA Pol II promoter, but not a polyadenylation site, seems to be essential for the inhibition of the NS1 suppression of reporter gene expression.

Recently, it was reported that IAV infection induces a defect in RNA Pol II transcription termination (10–12). Our RNASeq experiments demonstrate that NS1 effector domain is responsible for the deregulation of host transcriptional termination in IAV-infected cells. We further confirmed these data using a reporter gene assay. Most likely, both the NS1-mediated transcriptional inhibition described here and the defects in transcriptional termination are two sides of the same coin. Our observation that the NS1 effector domain mediates transcriptional inhibition and terminal deregulation are in line with two of the above-mentioned publications (10, 11). However, Bauer et al. (12) claim that cellular stress due to the virus infection induces the termination defect and that this defect is independent of the direct interaction between the viral NS1 protein and CPSF30. Heinz et al. (10) and Zhao et al. (11) found in agreement with our results that a gross deletion of the C terminus of NS1 or a deficiency of NS1, respectively, leads to a loss of the transcriptional termination defects similar to what we observed with the NS1 184-188 mutant IAV. Bauer et al. (12) could not detect a failure in transcription termination using C-terminally truncated NS1 of the subtype IAV/Udorn. These findings raise speculation that IAV subtypes might have evolved different strategies to modulate 3′-end formation due to subtle differences in binding strength and/or specificity of the strain-specific NS1 proteins to host transcription factors (10).

Since we found that the NS1 mutant 184-188 being unable to bind CPSF30 (15) also fails to mediate transcriptional inhibition, we tried to further clarify the role of CPSF30 in this process. CPSF30 overexpression clearly counteracts NS1-mediated transcriptional inhibition, which seems to support the longstanding hypothesis that CPSF30 is directly involved in this NS1 function. The hypothesis that the cellular availability of CPSF30 limits the transcriptional activity of the reporter genes and may be decreased by massive expression of NS1 via NS1-CPSF30 interaction could not be confirmed. In our hands, the siRNA-mediated knockdown of CPSF30 did not result in an inhibition of host transcription, since siRNA-mediated depletion of CPSF30 by siRNA did not reduce reporter gene activity. However, the knockdown of CPSF30 reportedly induces a RNA Pol II termination defect (12). In agreement with the latter finding is the observation that depletion of another protein of the CPSF complex, CPSF73, also induced RNA Pol II termination defects (10). Thus, our finding raises some doubts whether the NS1-CPSF30 interaction is essential for transcriptional inhibition as it is the case for the deregulation of termination and whether both NS1-mediated transcriptional inhibition and NS1-mediated deregulation of transcriptional termination mechanistically correlate. However, we cannot exclude that in our experiments some residual CPSF30 expression sustains CPSF30 function.

Furthermore, we could observe that nuclear wild-type NS1 is associated with insoluble chromatin according to the biochemical fractionation experiments. Surprisingly, this chromatin association is independent of the ability of NS1 to interact with CPSF30 and independent of the ability of NS1 to inhibit transcription as shown with the NS1 184-188 mutant but dependent on a functional RNA binding domain of NS1.

Our observations also argue against the recent findings of Anastasina et al. (18), who identified NS1 as a DNA-binding protein, which thereby blocks transcription of antiviral genes. Analysis by STORM microscopy does not indicate a major colocalization of NS1 and genomic DNA, which would be expected, if NS1 binds genomic DNA. The observed strong chromatin association of NS1 according to the fractionation and to the STORM microscopy data obviously relies on the RNA-binding domain. The mutant NS1 38, 41 and also the RNA-binding domain deletion mutant NS1 79-230 are both unable to bind to RNA and unable to bind to nuclear chromatin according to our cellular fractionation and STORM experiments. They are at least partially localized in the cytosol according to our gfp fusion protein data. Nonetheless, they are both still able to inhibit gene expression. Of note, in this context we define “chromatin localization” according to the criteria of the biochemical fractionation. Functionally, NS1 that mediates transcriptional shutoff is obviously located at the sites of transcription and splicing. The shutoff of gene expression despite the shift from nuclear to cytosolic NS1 implies that the presence of only a fraction of the expressed NS1 at the site of action is sufficient for the shutoff.

The data obtained with the quadruple mutant NS1 38, 41, 219, 220 indicated that amino acids 219 and 220 of the effector domain are—in contrast to 184-188—not essential for NS1-mediated host shutoff function but more likely for guiding NS1 to the correct site of action. The data led us to speculate that a functional effector domain of NS1 is sufficient to inhibit host gene expression and terminal deregulation when it is located in specific structures of the nucleus that resemble nuclear speckles. Interestingly, influenza B virus NS1 has been shown to accumulate in nuclear speckles independent of other viral functions (33). Since nuclear speckles are the site of mRNA transcription and maturation, this observation would fit to our data and to the finding of others that IAV infection provokes massive alterations in host gene transcription as a decreased Pol II occupancy downstream of transcription start sites as well as defects in termination (10–12).

## MATERIALS AND METHODS

### Cells, plasmids, viruses, chemicals and antibodies, and siRNAs.

Cells and vectors used to stably express the inducible NS1ERT are described in Nacken et al. (20). Reporter plasmids pHrD-IRES-luc, pTert-luc, and pCL-neo-mcherry-SRSF2 were a kind gift of S. T. Jacob, of Xiaonan Cui, Wuguo Deng, and Ge Liu (Dalian, China), and of N. Gehring (Cologne, Germany), respectively. The expression plasmids encoding T7 polymerase, pAR3132 and pAR3126, were both a kind gift of W. Studier (NY, USA).

All NS1‐coding sequences used in this study originate from seal influenza virus strain SC35M (H7N7), which is a mouse-adapted virus originally isolated from seals, but of avian origin. Recombinant SC35M IAV was generated using the eight-plasmid method based on pHW2000 vector (34). To enable the generation of any mutation in NS1 without interfering with the expression of overlapping NS2, we constructed a NS segment that separates the expression of NS1 and NS2 gene by a 2A peptide (18, 20).

PCR and cloning of mutant NS1 were performed according to standard methods. Transient overexpression of NS1 was performed by cloning the corresponding orfs into pCDN3 by standard cloning techniques. The expression levels of all expression plasmids have been controlled by Western blotting. To be sure to maintain the function of NS1 fusion proteins used in fluorescence microscopy, some of the NS1 fusion proteins (NS1 38,41-H2B, NS1 38,41-tubulin) were constructed as fusions with the GFP-11 domain and transfected in cells transduced with pQCXIP-gfp1-10 (Addgene no. 68715), resulting in fluorescence when GFP11 fusion protein and gfp1-10 complement each other by self-assembly (split gfp assay).

Commercially available antibodies were used to detect estrogen receptor ER alpha (Cloud Clone Corp., USA) or histidine-tagged (His-tag) proteins (ThermoFisher, Germany). Polyclonal NS1 (geneTex, USA) antibody was used to detect IAV NS1, and monoclonal CPSF4 antibody was obtained from Santa Cruz (sc-393316). RT-PCR primers and siRNAs used in this study are summarized in the supplemental material (see Tables S1 and S2 in the supplemental material).

### Luciferase assays.

Luciferase assays were performed as described by Nacken et al. (20). The relative light unit (RLU) values of the tamoxifen‐incubated cells were set in relation to RLU values derived from the nontreated cells.

### Cellular fractionation.

Cellular fractionation and chromatin isolation were essentially performed according to the protocol of Chase et al. (35). Sodium dodecyl sulfate (SDS)-polyacrylamide gel electrophoresis and Western blotting have been described earlier (20).

### Stochastic optical reconstruction microscopy (STORM) and fluorescence microscopy.

Cells were seeded into cell culture wells and treated with ethynyl-deoxyuridine (EdU) (1 μM, overnight) and then labeled by click chemistry according to the manufacturer’s protocol (Jena Bioscience). STORM microscopy was performed as described by Schreiber et al. (36). Briefly, STORM sample preparation was done according to the reporter-only method with conventional antibodies according to the Nikon N-STORM protocol for immune staining. Imaging of the samples was done with the N-STORM Ti-LAPP Ti laser application system. The calculation of the image reconstruction was done with NIS-Element Advanced Research – Imaging software (V4.51.01) using algorithms for molecule identification and drift correction. Fluorescence microscopy was performed according to standard methods. Cells were analyzed using Zeiss Confocal LSM800 microscope using Zen Lite system with the Plan-Apochromat 40×/1.40 oil differential contrast (DIC) M27 objective and AiryScan GaAsP detector (1 Airy unit [AU]). Pictures were prepared with Fiji/ImageJ software version 1.51n.

### RNA isolation and qRT-PCR.

Transcription of nascently transcribed RNA was analyzed in OHT-treated and nontreated NS1ERT-expressing cells. One hour after OHT induction, ethynyl-UTP was added to the medium for 2 h to label nascently transcribed RNA. Total RNA was then isolated with TRIzol following the manufacturer’s protocol. Subsequently, the labeled RNA was biotinylated via click-it chemistry, the biotinylated RNA was purified by streptavidin-coupled magnetic beads and finally reverse transcribed into DNA following the manufacturer’s protocol (Click-it Nascent RNA Capture kit; Thermosphere).

Given that transcription is globally affected by NS1, the transcription of the so-called housekeeping genes that are normally used as internal reference genes may also be affected by NS1. Therefore, we assumed that there is possibly no reliable internal cellular reference gene available for relative quantifications. Thus, we generated ethynyl-labeled RNA encoding luciferase by adding ethynyl-UTP in an *in vitro* T7 RNA polymerase reaction and transfected the T7 RNA into the NS1ERT-expressing cells before they were seeded into OHT-treated and nontreated populations. Luciferase cDNA was then used as an “internal” standard, whose transcription/concentration in the cell is not affected by any potential transcriptional inhibition of NS1ERT. For primer sequences see Table S1.

### Labeling RNA and FACS analysis.

Cellular RNA was labeled by adding ethynyl uridine (EU) to the medium. Subsequently, the cells were fixed and permeabilized with 4% paraformaldehyde and 0.1% Triton X-100. Chemical labeling of the EU with a fluorophore-containing RNA was performed by click chemistry according to the manufacturer’s protocol (Jena Bioscience, Germany). Cells were analyzed by FACS analysis according to standard procedures.

### Ranse experiment.

A549 cells were infected with wild-type IAV and IAV NS1 184-188 (both multiplicity of infection [MOI] of 5). Six hours postinfection (p.i.), total RNA was isolated. The library preparation of the total RNA (depleted for rRNA) was performed with the NEBNext Ultra II RNA directional kit, and single read sequencing was performed using a NextSeq 500 System with a read length of 75 bp. Using a molecular barcode, the samples will be demultiplexed (bcl2fastq2) to fastq data and quality controlled (FastQC). Trimmomatic will be used for adapter trimming and read filtering. The resulting reads were aligned to the Ensembl GRCh38 reference genome using Hisat22. The aligned reads were sorted using samtools3. The sorted and aligned reads were counted into genes using htsec-counts 4a. The IGV software (integrated genome viewer; software from the Broad Institute) was used to view the aligned Illumina reads to the human genome and to visualize the data.

### Data availability.

Sequencing data have now been deposited under https://www.ncbi.nlm.nih.gov/Traces/study/?acc=PRJNA748585; BioSample accession numbers SAMN20339638 and SAMN20339639.
