# Supplementary material for: The Effector Domain of the Influenza A Virus Nonstructural Protein NS1 Triggers Host Shutoff by Mediating Inhibition and Global Deregulation of Host Transcription When Associated with Specific Structures in the Nucleus
Source: mBio. 2021 Sep 7;12(5):e02196-21. doi: 10.1128/mBio.02196-21 (PMC8546537; doi:10.1128/mBio.02196-21)
Supplement: TABLE S1 [file mbio.02196-21-st001.docx]

**Supplementary material**

Table 1

Primers used for RT-PCR

| Gene | forward pimer | Reverse primer |
| --- | --- | --- |
| luciferase | agagatcctcataaa | atttgtattcagcccatatcgttt |
| hsp90 | gcttgaccaatgactgggaag | agctcctcacagttatccatga |
| gapdh | ctgggctacactgagcacc | aagtggtcgttgagggcaatg |
| rpl3 | ctaccatcaccgcactgagat | ggtcacttcaccatagtggaca |
| Telomerase | tgtgcaccaacatctacaagatcc | ctgatgaaatgggagctgacg |
| CPSF30 | tgtccgtttcgccacatcag | ttggtcatgtcatactcatgcag |
| 5S RNA | ggccataccaccctgaacgc | cagcacccggtattcccagg |
| tRNA thr | gtggccaagtggtaaggcgtc | acccgacttccccacagcc |
| alu | acgcctgtaatcccagcactt | tcgcccaggctgggtgca |
| 18S RNA (P) | gcttaatttgactcaaca | agctatcaatctgtcaat |
| 18S RNA (N) | gtaacccgttgaaccccatt | ccatccaatcggtagtagcg |
| NS1 | aattgtcggcgaaatttcac | tgactcaattgttctcgcca |
