# Supplementary material for: The Effector Domain of the Influenza A Virus Nonstructural Protein NS1 Triggers Host Shutoff by Mediating Inhibition and Global Deregulation of Host Transcription When Associated with Specific Structures in the Nucleus
Source: mBio. 2021 Sep 7;12(5):e02196-21. doi: 10.1128/mBio.02196-21 (PMC8546537; doi:10.1128/mBio.02196-21)
Supplement: TABLE S2 [file mbio.02196-21-st002.docx]

**Supp. Table 2**

Si RNAs CPSF30:

esiRNA Nr. EHU132701 (Sigma)

S21412 (Exon3) (ThermoFisher)

S21411 (Exon 7/8) (ThermoFisher)

siRNA: caugcacccucgauuugaatt (Eurofins)*

siRNA: ggucaccuguuacaagugutt (Eurofins)*

*both sequences were extracted from:

Chen W, Guo W, Li M, Shi D, Tian Y, Li Z2, Wang J, Fu L, Xiao X, Liu QQ, Wang S, Huang W, Deng W. Upregulation of cleavage and polyadenylation specific factor 4 in lung adenocarcinoma and its critical role for cancer cell survival and proliferation. PLoS One. 2013 Dec 16;8(12):e82728. doi: 10.1371/journal.pone.0082728. eCollection 2013.
